# Effects of therapies for Ebola virus disease: a systematic review and network meta-analysis

**DOI:** 10.1016/S2666-5247(22)00123-9

**Published:** 2022-09

**Authors:** Ya Gao, Yunli Zhao, Gordon Guyatt, Robert Fowler, Richard Kojan, Long Ge, Jinhui Tian, Collaborators, Janet Diaz, Marta Lado, Daniel Youkee, Aasim Ahmad, Cindy Albertson, Séverine Caluwaerts, Modet Camara, Ian Crozier, Hilde De Clerck, Susanna Dunachie, William A Fischer, Bushra Jamil, Patrice Kabongo, Patricia Kabuni, Charline Kahambu Ngorombi, Maurice Kakule, Marie-Claire Kolié, Sulaiman Lakoh, Hans-Jörg Lang, J Soka Moses, Isekusu Mpinda Fiston, Philippe Mukumbayi Mulumba, Srinivas Murthy, Sorie Samura, Rachel Couban, Qiukui Hao

**Affiliations:** a Evidence-Based Medicine Center, School of Basic Medical Sciences, Lanzhou University, Lanzhou, China; b Evidence-Based Social Science Research Center, School of Public Health, Lanzhou University, Lanzhou, China; c Department of Health Research Methods, Evidence, and Impact, McMaster University, Hamilton, ON, Canada; d Department of Medicine, McMaster University, Hamilton, ON, Canada; e School of Rehabilitation Science, McMaster University, Hamilton, ON, Canada; f National Clinical Research Center for Geriatrics, West China Hospital, Sichuan University, Chengdu, China; g Interdepartmental Division of Critical Care Medicine, University of Toronto, Toronto, ON, Canada; h Institute of Health Policy, Management and Evaluation, Dalla Lana School of Public Health, University of Toronto, Toronto, ON, Canada; i Department of Critical Care Medicine, Sunnybrook Health Sciences Centre, Toronto, ON, Canada; j Alliance for International Medical Action, Dakar, Senegal

## Abstract

**Background:**

Specific treatments targeting Ebola virus are crucial in managing Ebola virus disease. To support the development of clinical practice guidelines on medications for Ebola virus disease, we aimed to evaluate the efficacy and safety of therapies for patients with Ebola virus disease.

**Methods:**

In this systematic review and network meta-analysis, we searched MEDLINE, Embase, the Cochrane Central Register of Controlled Trials, Scopus, Global Health, African Index Medicus, World Health Organization Global Index Medicus, the Cumulative Index to Nursing and Allied Health Literature, ClinicalTrials.gov, Epistemonikos, bioRxiv, medRxiv, and SSRN without language restrictions for randomised controlled trials (RCTs) published between database inception and Jan 1, 2022, comparing at least one therapeutic agent for Ebola virus disease against standard care or another therapeutic agent for Ebola virus disease. Two reviewers assessed study eligibility and extracted summary data independently using a standardised form. Our outcomes of interest were mortality, adverse maternal outcomes, risk of onward transmission, duration of admission to a health-care facility, functional status after Ebola virus disease, serious adverse events from medication, adverse perinatal outcomes, time to symptom resolution, and time to viral clearance. We did frequentist network meta-analyses to estimate the effect of all interventions and applied the Grading of Recommendations Assessment, Development and Evaluation approach to rate the certainty of the evidence. We registered the protocol with PROSPERO, CRD42022296539.

**Findings:**

We identified 7840 records through database searches, of which two RCTs with a total of 753 patients proved eligible. Only data on mortality, the duration of admission, serious adverse events, and time to viral clearance were available for meta-analysis. Compared with standard care, REGN-EB3 (relative risk [RR] 0·40, 95% CI 0·18 to 0·89; moderate certainty) and mAb114 (0·42, 0·19 to 0·93; moderate certainty) probably reduce mortality. Whether ZMapp (0·60, 0·28 to 1·26; very low certainty) and remdesivir (0·64, 0·29 to 1·39; very low certainty) reduce mortality compared with standard care is uncertain. With high certainty, REGN-EB3 reduces mortality compared with ZMapp (0·67, 0·52 to 0·88) and remdesivir (0·63, 0·49 to 0·82). With high certainty, mAb114 also reduces mortality compared with ZMapp (0·71, 0·55 to 0·91) and remdesivir (0·66, 0·52 to 0·84). Compared with standard care, REGN-EB3, mAb114, ZMapp, and remdesivir might have little or no effect on the time to viral clearance (mean difference ranged from –0·25 days to –1·14 days; low certainty). ZMapp might reduce the duration of admission compared with standard care (mean difference –2·02 days, 95% CI –4·05 to 0·01; low certainty). Findings for all comparisons suggested that there might be little or no difference in the prevalence of serious adverse events, but certainty was low or very low in all comparisons but one.

**Interpretation:**

REGN-EB3 and mAb114 separately reduce mortality compared with ZMapp, remdesivir, or standard care in patients with Ebola virus disease. These findings suggest that health-care workers should prioritise the use of REGN-EB3 and mAb114 for patients with Ebola virus disease during future outbreaks.

**Funding:**

WHO.

## Introduction

Ebola virus disease is a severe acute infectious disease with high mortality. Among Ebola outbreaks between 2010 and 2021, mortality ranged from 25% to 90%.^1^ Early case finding, vaccination, and using the best supportive care—including rehydration with oral or intravenous fluids and the treatment of specific symptoms or complications—can improve survival.^2^ Nevertheless, mortality from Ebola virus disease remained high (≥50%) in outbreaks in the Democratic Republic of the Congo and Guinea in 2021.^3^ Additional specific treatments targeting Ebola virus therefore remain a crucial element of managing Ebola virus disease.

By use of data from 35 studies, most of which were limited by suboptimal non-randomised study designs and small sample sizes, a previous systematic review^4^ evaluated the effect of 21 anti-Ebola virus therapies (eg, ZMapp, favipiravir, brincidofovir, and convalescent plasma). The review identified only one randomised controlled trial (RCT)^5^ of an anti-Ebola virus therapy (ZMapp) and found that ZMapp might be beneficial in reducing mortality compared with standard care (absolute risk reduction 15%, 95% CI –7% to 36%). In 2019, a new RCT^6^ reported that mAb114 and REGN-EB3 were superior to ZMapp in reducing mortality for patients with Ebola virus disease, highlighting a need to systematically review all available RCTs.

**Research in context**

**Evidence before this study**

WHO's evidence-based clinical practice guidelines published in 2018 focus only on the delivery of optimised supportive care for patients with Ebola virus disease. So far, no guidelines have focused on specific therapies for Ebola virus disease. Previous systematic reviews with small sample sizes have reported that an Ebola-virus-specific treatment (ZMapp) might have a treatment effect. New evidence emerged in 2019 for other drugs, including mAb114 and REGN-EB3. The relative merits of these drugs remain unclear. We searched MEDLINE, Embase, the Cochrane Central Register of Controlled Trials, Scopus, Global Health, African Index Medicus, World Health Organization Global Index Medicus, the Cumulative Index to Nursing and Allied Health Literature, ClinicalTrials.gov, Epistemonikos, bioRxiv, medRxiv, and SSRN without language restrictions from database inception to Jan 1, 2022. Search terms included “ebola virus disease” and “randomized controlled trials”. We included randomised clinical trials that compared at least one therapeutic agent for Ebola virus disease against standard care or another therapeutic agent for Ebola virus disease.

**Added value of this study**

To our knowledge, this systematic review and network meta-analysis is the first to directly and indirectly evaluate the efficacy and safety of different therapies for patients with Ebola virus disease. We summarise the evidence comparing four therapies (REGN-EB3, mAb114, ZMapp, and remdesivir) versus standard care and one another, and present absolute estimates of effects and the certainty of evidence across treatment options. This study shows that REGN-EB3 and mAb114 separately reduce mortality compared with ZMapp or remdesivir and that REGN-EB3 and mAb114 probably reduce mortality relative to standard care. Whether ZMapp and remdesivir reduce mortality compared with standard care is very uncertain. There is no convincing difference in the prevalence of serious adverse events between REGN-EB3, mAb114, ZMapp, remdesivir, and standard care.

**Implications of all the available evidence**

Our study provides evidence that REGN-EB3 and mAb114 are separately superior to ZMapp, remdesivir, or standard care in reducing mortality among patients with Ebola virus disease. These findings suggest that health-care workers should prioritise the use of REGN-EB3 and mAb114 for patients with Ebola virus disease during future outbreaks.

Evidence-based clinical practice guidelines published by WHO in 2018 focus only on the delivery of optimised supportive care for patients with Ebola virus disease.^7^ So far, no guidelines have focused on specific medication therapies for Ebola virus disease. Thus, WHO formulated an international guideline panel (WHO Guideline Development Group for Therapeutics for Ebola Virus Disease) to develop, on the basis of all available evidence from RCTs, a clinical practice guideline addressing treatment for Ebola virus disease. To support the development of the new guideline, we aimed to do a systematic review and network meta-analysis of RCTs to evaluate the efficacy and safety of therapies for patients with Ebola virus disease.

## Methods

### Search strategy and selection criteria

We report this systematic review and network meta-analysis according to the Preferred Reported Items for Systematic Reviews and Meta-Analyses statement for network meta-analyses.^8^ With the aid of a medical librarian, we searched MEDLINE, Embase, the Cochrane Central Register of Controlled Trials, Scopus, Global Health, African Index Medicus, World Health Organization Global Index Medicus, the Cumulative Index to Nursing and Allied Health Literature, ClinicalTrials.gov, Epistemonikos, bioRxiv (preprints), medRxiv (preprints), and SSRN (preprints) without language restrictions from database inception to Jan 1, 2022. The search terms included “ebola virus disease” and “randomized controlled trials” and can be found in full in the appendix (pp 3–7). We also manually searched the reference lists of relevant systematic reviews, eligible studies, and related articles.

Eligible RCTs enrolled patients with confirmed Ebola virus disease (by RT-PCR) according to WHO definitions^9^ caused by any species of Ebola virus and compared at least one therapeutic agent for Ebola virus disease against standard care alone or another therapeutic agent for Ebola virus disease. We defined therapeutic agents for Ebola virus disease as therapies specifically targeting Ebola virus itself or its clinical consequences (eg, anti-inflammatories, antifibrinolytics, and blood component-based strategies) and consulted with the guideline panel if classification issues arose. Eligible trials reported at least one of the outcomes of interest. We did not apply restrictions on the severity of illness or the age of patients. We excluded studies investigating vaccines for the primary prevention of Ebola virus disease, therapies for viral persistence in survivors of Ebola virus disease, or agents targeting postexposure prophylaxis.

Two reviewers (YG and QH) conducted the literature searches. Two reviewers (YG and YZ) assessed study eligibility and the risk of bias, and extracted data independently, resolving disagreements by discussion and, if necessary, adjudication by a third reviewer (QH). Reviewers screened the titles and abstracts of identified records and then retrieved the full texts of potentially eligible records to further assess their eligibility. We used Covidence for screening. For missing or unclear data, we contacted authors for further information about summary estimates. The study protocol can be found online.

### Data analysis

Reviewers independently extracted summary estimate data using a standardised form that comprised the following variables: study characteristics (first author, trial registration, publication status, study status, and design); patient characteristics (country of patient recruitment, sample size, age, sex distribution, comorbidities, and severity of Ebola virus disease); characteristics of interventions and comparators (doses, dose schedule, route of administration, treatment duration, details of standard of care, and length of follow-up); and data on each outcome of interest.

Our methods for selecting outcomes of interest can be found in the appendix (pp 8–9). The outcomes of interest we selected were mortality; adverse maternal outcomes (eg, antepartum and post-partum haemorrhage, obstructed labour, hypertensive disorders, and maternal sepsis); the risk of onward transmission; the duration of admission (time from admission at a health-care facility to the discharge date for survivors); functional status after Ebola virus disease (ability to perform activities of daily living); serious adverse events (associated with the use of medication); adverse perinatal outcomes (eg, preterm birth, low birthweight, stillbirth, and neonatal death); time to symptom resolution (time from the first reported symptom to the first day of being symptom-free); and time to viral clearance (time from the first RT-PCR test positive for Ebola virus to the first RT-PCR test negative for Ebola virus).

Comparisons of medications in this Article are of each medication added to optimised supportive care (standard care) compared with either other medications plus standard care or optimised supportive care alone. For simplicity, we subsequently refer to these comparisons as a particular medication directly or indirectly compared with standard care.

For the outcomes of time to viral clearance and duration of admission, the eligible trials did not report data on variability (eg, SD, 95% CI, or IQR) to allow for meta-analysis. We requested data from the authors of the eligible trials; the authors provided medians and 95% CIs for mAb114 and REGN-EB3, but did not provide data on variability for ZMapp and remdesivir that would allow for analysis. Therefore, for these outcomes, we obtained data from the figures included in the eligible studies for survivors and used these data for analysis.

We did pairwise meta-analyses in R, version 3.6.3, for all direct comparisons of each outcome. Regarding dichotomous outcomes, we calculated relative risks (RRs) with 95% CIs for mortality and risk differences with 95% CIs for serious adverse events. We calculated mean differences with 95% CIs for continuous outcomes.

To describe and present the geometry of the network of comparisons across trials, we used STATA, version 15.0, to draw network plots. We did frequentist network meta-analyses to estimate the effect of all interventions using the netmeta (version 1.3-0) statistical package in R, version 3.6.3. We used the side-splitting method to obtain indirect estimates.^10^ We present direct, indirect, and network estimates for outcomes with available data (for further details of these analyses, see appendix pp 10–11).

To facilitate interpretation of the results, we used network RR estimates and the baseline risk to calculate absolute effects for mortality, time to viral clearance, and duration of admission. For mortality, due to its large variability, we estimated two baseline risks: the lowest and highest mortalities in outbreaks with at least 100 diagnosed patients since 2013 reported by WHO's website.^11^ For time to viral clearance and the duration of admission, we used the median in the standard care group from eligible RCTs as the baseline mean.

When data were available, we did prespecified analyses in the following subgroups: patient age (≤5 years *vs* 6–59 years *vs* ≥60 years); previous vaccination against Ebola virus disease (<10 days *vs* ≥10 days relative to randomisation); duration of symptoms before treatment (≤5 days *vs* >5 days); pregnancy (pregnant *vs* non-pregnant); and baseline cycle threshold (Ct) value (a value used to measure Ebola virus RNA concentrations; Ct ≤22 *vs* Ct >22; appendix pp 10–11). The Ct cutoff threshold was chosen after discussions in a consensus meeting and was based on current evidence, the threshold chosen in the eligible trials, and the perception of clinical experts. We assessed the credibility of possible subgroup analysis using the Instrument for assessing the Credibility of Effect Modification Analyses tool.^12^

When studies reported missing outcome data, we did a complete case analysis as our primary analysis. To assess the impact of missing outcome data, we did a prespecified plausible worst-case sensitivity analysis to evaluate the robustness of the results.^13^ The paucity of studies meant we could not assess between-study heterogeneity and publication bias.

We used the Grading of Recommendations Assessment, Development and Evaluation (GRADE) approach for network meta-analysis to assess the certainty of the direct, indirect, and network estimates for outcomes with available data.^14^, ^15^ We used a minimally contextualised approach and rated certainty according to an effect greater or less than the minimally important difference.^16^, ^17^ We rated, by assessing domains of the risk of bias, inconsistency, indirectness, imprecision, and publication bias,^18^, ^19^, ^20^, ^21^, ^22^, ^23^, ^24^, ^25^ the certainty of evidence for each comparison and outcome as high, moderate, low, or very low. We assessed imprecision at the network level by comparing CIs to thresholds^21^ agreed upon by the guideline panel for each outcome. The minimally important difference threshold was 1% for mortality and 2% for serious adverse events and 1 day for time to viral clearance and duration of admission; we followed GRADE guidance for rating down for imprecision.^26^ We used the MAGICapp platform to develop the GRADE summary of findings tables.^27^ For further details of GRADE ratings of certainty, see the appendix (pp 12–13). Two reviewers (YG and YZ) assessed the risk of bias of included RCTs using a modified Cochrane risk of bias tool (appendix p 14).^28^ We registered the protocol for this systematic review and network meta-analysis with PROSPERO, CRD42022296539.

### Role of the funding source

The funder of the study had no role in study design, data collection, data analysis, data interpretation, or writing of the report.

## Results

**Figure 1:**
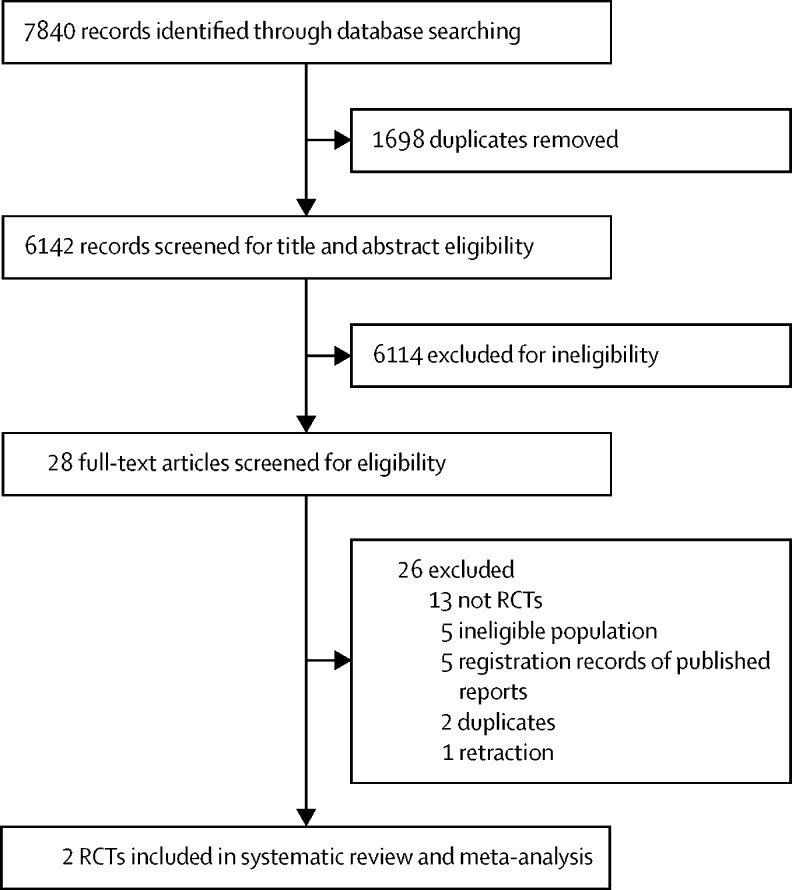
Study selection RCTs=randomised controlled trials.

We identified 7840 records through electronic database searches, of which two RCTs^5^, ^6^ proved eligible after screening (figure 1). These two trials,^5^, ^6^ published in 2016 and 2019, enrolled 753 patients in total (table 1). The PREVAIL II trial,^5^ which was done in Guinea, Liberia, Sierra Leone, and the USA, compared ZMapp with standard care and randomly assigned 72 patients. The mean age of patients was 26·1 years (SD 17·4), the mean Ct value was 23·9 (5·3), and the mean duration of symptoms at baseline was 4·2 days (2·7). The PALM trial^6^ was done in the Democratic Republic of the Congo and randomly assigned 681 patients to receive standard care plus either ZMapp, remdesivir, mAb114, or REGN-EB3. The mean age of patients was 28·8 years (17·6), the mean Ct value was 24·0 (5·6), and the mean duration of symptoms at baseline was 5·5 days (3·5). The two trials^5^, ^6^ specified similar standard-of-care management, generally including the administration of intravenous fluids, laboratory testing, the correction of hypoglycaemia and electrolyte imbalances, and the administration of concomitant medications (appendix p 15); however, standard-of-care management was not strictly protocolised and probably varied substantially across treatment units and time.

**Table 1:** Characteristics of included studies

	**Country or countries**	**Study design**	**Number of patients randomly assigned**	**Mean age, years**	**Sex**		**Pregnancy test positive in female patient**	**Mean Ct value at baseline**	**Proportion with Ct value ≤22 at baseline**	**Mean duration of symptoms at baseline, days**	**Overall mortality**	**Treatment doses and schedules**	**Outcomes**
					Male	Female							
PREVAIL II; Davey et al (2016)^5^	Guinea, Liberia, Sierra Leone, and the USA	Two treatment arms (ZMapp *vs* standard care)	72	26·1 (17·4)	32/72 (44%)	40/72 (56%)	0/40	23·9 (5·3)	30/72 (42%)	4·2 (2·7)	21/71 (30%)	ZMapp (50 mg/kg of bodyweight every third day for a total of three doses) and standard care	Mortality; serious adverse events; time to viral clearance; andduration of admission
PALM; Mulangu et al (2019)^6^	Democratic Republic of the Congo	Four treatment arms (ZMapp *vs* remdesivir *vs* mAb114 *vs* REGN-EB3)	681	28·8 (17·6)	299/673 (44%)	374/673 (56%)	17/374 (5%)	24·0 (5·6)	282/670 (42%)	5·5 (3·5)	290/673 (43%)	ZMapp (50 mg/kg of bodyweight every third day beginning on day 1 for a total of three doses); remdesivir (loading dose on day 1 [200 mg in adults and adjusted for bodyweight in children], followed by a daily maintenance dose [100 mg in adults and adjusted for bodyweight in children] starting on day 2 and continuing for 9–13 days, depending on viral load); mAb114 (single infusion of 50 mg/kg on day 1); and REGN-EB3 (single infusion of 150 mg/kg on day 1)	Mortality; serious adverse events; andtime to viral clearance

Data are mean (SD) or n/N (%). Ct=cycle threshold.

**Figure 2:**
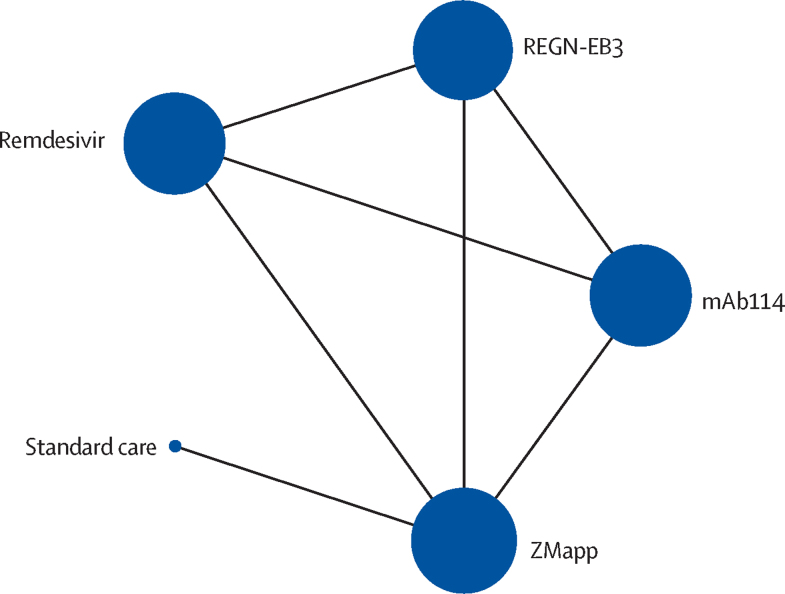
Network plot for mortality The size of the circle represents the number of participants. The connecting lines represent direct comparisons. The width of the line represents the number of studies.

We present the network plot for mortality (figure 2). All other network plots are in the appendix (pp 32–33). We present direct, indirect, and network estimates for each comparison for the outcomes of mortality (appendix p 16), serious adverse events (appendix p 17), and time to viral clearance (appendix p 18); only one study reported on the duration of admission between ZMapp and standard care (direct evidence) and there were no data for other comparisons. Since there was only direct or indirect evidence available for each comparison, tests of incoherence were not applicable for all network meta-analyses. Due to the small number of pregnant participants enrolled in the PALM trial (n=18), as reported by the study authors, we could not conduct meta-analytic or subgroup analyses on adverse maternal and perinatal outcomes (appendix p 19). Moreover, no trials evaluated therapies for the outcomes of risk of onward transmission, functional status after Ebola virus disease, and time to symptom resolution, and so data for these are not reported here.

We present the GRADE summary of findings for mortality (table 2), serious adverse events (table 3), time to viral clearance (appendix p 20), and duration of admission (appendix p 21). We judged the certainty of evidence to be low or very low for many comparisons because of the high risk of bias and serious imprecision. In the two studies,^5^, ^6^ mortality data at 28 days' follow-up were available for 744 patients. Network meta-analysis showed that, compared with standard care, REGN-EB3 (RR 0·40, 95% CI 0·18–0·89; absolute risk difference varied from –237 per 1000 patients to –396 per 1000 patients) and mAb114 (RR 0·42, 0·19–0·93; absolute risk difference varied from –229 per 1000 patients to –383 per 1000 patients) probably reduce mortality (table 2). Compared with standard care, whether ZMapp (RR 0·60, 0·28–1·26) and remdesivir (RR 0·64, 0·29–1·39) reduce mortality is very uncertain (table 2). High-certainty evidence showed that REGN-EB3, as well as mAb114, reduce mortality relative to ZMapp or remdesivir (table 2). Due to the paucity of trials and on the basis of the data obtained, we made some amendements to the prespecified subgroup analyses. We changed the age groups to 5 years or younger, 6–17 years, and 18 years or older, vaccination duration to previously vaccinated versus unvaccinated, and the duration of symptoms at baseline to 4 days or fewer versus more than 4 days. Subgroup analyses of mortality mostly did not reveal any subgroup effects, except for effect modification by Ct value (≤22 *vs* >22) for comparisons of mAb114 versus ZMapp or remdesivir (appendix pp 22–25). However, the credibility of these subgroup effects was rated as low because we did multiple subgroup tests, increasing the possibility of a chance-related finding (appendix pp 26–29). Data from two studies^5^, ^6^ of 744 patients were included in the serious adverse event analysis. Findings for all comparisons suggested that there was little or no difference in the prevalence of serious adverse events, but certainty was low or very low in all comparisons but one (table 3).

**Table 2:** GRADE summary of findings for mortality for different comparisons

		**Study results and measurements**	**Absolute effect estimates per 1000 patients**		**Absolute difference per 1000 patients (95% CI)**	**Certainty in effect estimates**	**Plain language summary**
			Treatment 1	Treatment 2			
REGN-EB3 versus standard care		Relative risk 0·40 (95% CI 0·18 to 0·89); based on indirect evidence	..	..	..	..	..
	Absolute effects estimated from lowest baseline risk	..	158 (REGN-EB3)	395 (standard care)	−237 (−324 to −43)	Moderate*	REGN-EB3 probably reduces mortality compared with standard care when using the lowest baseline risk estimate
	Absolute effects estimated from highest baseline risk	..	264 (REGN-EB3)	660 (standard care)	−396 (−541 to −73)	Moderate*	REGN-EB3 probably reduces mortality compared with standard care when using the highest baseline risk estimate
	mAb114 versus standard care	Relative risk 0·42 (95% CI 0·19 to 0·93); based on indirect evidence	..	..	..	..	..
	Absolute effects estimated from lowest baseline risk	..	166 (mAb114)	395 (standard care)	−229 (−320 to −28)	Moderate*	mAb114 probably reduces mortality compared with standard care when using the lowest baseline risk estimate
	Absolute effects estimated from highest baseline risk	..	277 (mAb11)	660 (standard care)	−383 (−535 to −46)	Moderate*	mAb114 probably reduces mortality compared with standard care when using the highest baseline risk estimate
ZMapp versus standard care		Relative risk 0·60 (95% CI 0·28 to 1·26); based on data from 71 participants in one study	..	..	..	..	..
	Absolute effects estimated from lowest baseline risk	..	237 (ZMapp)	395 (standard care)	−158 (−284 to 103)	Very low†	Whether ZMapp reduces mortality compared with standard care is very uncertain when using the lowest baseline risk estimate
	Absolute effects estimated from highest baseline risk	..	396 (ZMapp)	660 (standard care)	−264 (−475 to 172)	Very low†	Whether ZMapp reduces mortality compared with standard care is very uncertain when using the highest baseline risk estimate
Remdesivir versus standard care		Relative risk 0·64 (95% CI 0·29 to 1·39); based on indirect evidence	..	..	..	..	..
	Absolute effects estimated from lowest baseline risk	..	253 (remdesivir)	395 (standard care)	−142 (−280 to 154)	Very low†	Whether remdesivir reduces mortality compared with standard care is very uncertain when using the lowest baseline risk estimate
	Absolute effects estimated from highest baseline risk	..	422 (remdesivir)	660 (standard care)	−238 (−469 to 257)	Very low†	Whether remdesivir reduces mortality compared with standard care is very uncertain when using the highest baseline risk estimate
REGN-EB3 versus mAb114		Relative risk 0·96 (95% CI 0·71 to 1·29); based on data from 329 participants in one study	..	..	..	..	..
	Absolute effects estimated from lowest baseline risk	..	159 (REGN-EB3)	166‡ (mAb114)	−7 (−48 to 48)	Low§	There might be little or no difference between REGN-EB3 and mAb114 when using the lowest baseline risk estimate
	Absolute effects estimated from highest baseline risk	..	266 (REGN-EB3)	277‡ (mAb114)	−11 (−80 to 80)	Low§	There might be little or no difference between REGN-EB3 and mAb114 when using the highest baseline risk estimate
REGN-EB3 versus ZMapp		Relative risk 0·67 (95% CI 0·52 to 0·88); based on data from 324 participants in one study	..	..	..	..	..
	Absolute effects estimated from lowest baseline risk	..	159 (REGN-EB3)	237¶ (ZMapp)	−78 (−114 to −28)	High	REGN-EB3 reduces mortality compared with ZMapp when using the lowest baseline risk estimate
	Absolute effects estimated from highest baseline risk	..	265 (REGN-EB3)	396¶ (ZMapp)	−131 (−190 to −48)	High	REGN-EB3 reduces mortality compared with ZMapp when using the highest baseline risk estimate
REGN-EB3 versus remdesivir		Relative risk 0·63 (95% CI 0·49 to 0·82); based on data from 330 participants in one study	..	..	..	..	..
	Absolute effects estimated from lowest baseline risk	..	159 (REGN-EB3)	253‖ (remdesivir)	−94 (−129 to −46)	High	REGN-EB3 reduces mortality compared with remdesivir when using the lowest baseline risk estimate
	Absolute effects estimated from highest baseline risk	..	266 (REGN-EB3)	422‖ (remdesivir)	−156 (−215 to −76)	High	REGN-EB3 reduces mortality compared with remdesivir when using the highest baseline risk estimate
mAb114 versus ZMapp		Relative risk 0·71 (95% CI 0·55 to 0·91); based on data from 343 participants in one study	..	..	..	..	..
	Absolute effects estimated from lowest baseline risk	..	168 (mAb114)	237¶ (ZMapp)	−69 (−107 to −21)	High	mAb114 reduces mortality compared with ZMapp when using the lowest baseline risk estimate
	Absolute effects estimated from highest baseline risk	..	281 (mAb114)	396¶ (ZMapp)	−115 (−178 to −36)	High	mAb114 reduces mortality compared with ZMapp when using the highest baseline risk estimate
mAb114 versus remdesivir		Relative risk 0·66 (95% CI 0·52 to 0·84); based on data from 349 participants in one study	..	..	..	..	..
	Absolute effects estimated from lowest baseline risk	..	167 (mAb114)	253‖ (remdesivir)	−86 (−121 to −40)	High	mAb114 reduces mortality compared with remdesivir when using the lowest baseline risk estimate
	Absolute effects estimated from highest baseline risk	..	279 (mAb114)	422‖ (remdesivir)	−143 (−203 to −68)	High	mAb114 reduces mortality compared with remdesivir when using the highest baseline risk estimate
ZMapp versus remdesivir		Relative risk 0·94 (95% CI 0·76 to 1·15); based on data from 344 participants in one study	..	..	..	..	..
	Absolute effects estimated from lowest baseline risk	..	238 (ZMapp)	253‖ (remdesivir)	−15 (−61 to 38)	Low§	There might be little or no difference between ZMapp and remdesivir when using the lowest baseline risk estimate
	Absolute effects estimated from highest baseline risk	..	397 (ZMapp)	422‖ (remdesivir)	−25 (−101 to 63)	Low§	There might be little or no difference between ZMapp and remdesivir when using the highest baseline risk estimate

GRADE=Grading of Recommendations Assessment, Development and Evaluation.

* Rated down for imprecision. Due to the superiority of REGN-EB3 and mAb114 over remdesivir and ZMapp and the low likelihood that REGN-EB3 or mAb114 increase mortality, we rated the certainty of evidence as moderate for these two drugs against standard care.

† Rated down three levels for imprecision.

‡ We used the point estimate of the absolute effect of mAb114, obtained from mAb114 versus standard care, to calculate the absolute effect for REGN-EB3 versus mAb114.

§ Rated down two levels for imprecision.

¶ We used the point estimate of the absolute effect of ZMapp, obtained from ZMapp versus standard care, to calculate the absolute effect for REGN-EB3 or mAb114 versus ZMapp.

‖ We used the point estimate of the absolute effect of remdesivir, obtained from remdesivir versus standard care, to calculate the absolute effect for REGN-EB3, mAb114, or ZMapp versus remdesivir.

**Table 3:** GRADE summary of findings for serious adverse events for different comparisons

	**Study results and measurements**	**Absolute difference per 1000 patients (95% CI)**	**Certainty in effect estimates**	**Plain language summary**
REGN-EB3 versus standard care	Risk difference 0·016 (95% CI −0·061 to 0·093); based on indirect evidence	16 (−61 to 93)	Very low*†	Whether REGN-EB3 increases the prevalence of serious adverse events compared with standard care is very uncertain
mAb114 versus standard care	Risk difference 0·016 (95% CI −0·061 to 0·093); based on indirect evidence	16 (−61 to 93)	Very low*†	Whether mAb114 increases the prevalence of serious adverse events compared with standard care is very uncertain
ZMapp versus standard care	Risk difference 0·028 (95% CI −0·046 to 0·102); based on data from 71 participants in one study	28 (−46 to 102)	Very low*†	Whether ZMapp increases the prevalence of serious adverse events compared with standard care is very uncertain
Remdesivir versus standard care	Risk difference 0·022 (95% CI −0·056 to 0·099); based on indirect evidence	22 (−56 to 99)	Very low*†	Whether remdesivir increases the prevalence of serious adverse events compared with standard care is very uncertain
REGN-EB3 versus mAb114	Risk difference 0·000 (95% CI −0·012 to 0·012); based on data from 329 participants in one study	0 (−12 to 12)	Moderate*	There is probably little or no difference between REGN-EB3 and mAb114 in the prevalence of serious adverse events
REGN-EB3 versus ZMapp	Risk difference −0·012 (95% CI −0·032 to 0·008); based on data from 324 participants in one study	−12 (−32 to 8)	Low*‡	There might be little or no difference in the prevalence of serious adverse events between REGN-EB3 and ZMapp
REGN-EB3 versus remdesivir	Risk difference −0·006 (95% CI −0·022 to 0·011); based on data from 330 participants in one study	−6 (−22 to 11)	Low*‡	There might be little or no difference in the prevalence of serious adverse events between REGN-EB3 and remdesivir
mAb114 versus ZMapp	Risk difference −0·012 (95% CI −0·032 to 0·008); based on data from 343 participants in one study	−12 (−32 to 8)	Low*‡	There might be little or no difference in the prevalence of serious adverse events between mAb114 and ZMapp
mAb114 versus remdesivir	Risk difference −0·006 (95% CI −0·021 to 0·010); based on data from 349 participants in one study	−6 (−21 to 10)	Low*‡	There might be little or no difference in the prevalence of serious adverse events between mAb114 and remdesivir
ZMapp versus remdesivir	Risk difference 0·006 (95% CI −0·017 to 0·029); based on data from 344 participants in one study	6 (−17 to 29)	Low*‡	There might be little or no difference in the prevalence of serious adverse events between ZMapp and remdesivir

GRADE=Grading of Recommendations Assessment, Development and Evaluation.

* Rated down for risk of bias.

† Rated down two levels for imprecision.

‡ Rated down for imprecision.

Data for time to viral clearance for survivors were available for 433 patients.^5^, ^6^ REGN-EB3, mAb114, ZMapp, and remdesivir might have little or no effect on the time to viral clearance compared with standard care (mean difference ranged from –0·25 days to –1·14 days; low certainty; appendix p 20). Compared with ZMapp or remdesivir, REGN-EB3 might (low certainty) and mAb114 probably does (moderate certainty) not reduce the time to viral clearance (appendix p 20). There is probably no substantial difference between REGN-EB3 and mAb114 (moderate certainty) and there might be little or no difference between ZMapp and remdesivir (low certainty) in time to viral clearance (appendix p 20). Data were available for 50 patients from one study^5^ for duration of admission. ZMapp might reduce the duration of admission compared with standard care (mean difference –2·02 days, 95% CI –4·05 to 0·01; low certainty; appendix p 21).

We judged all studies to be at low risk of bias in all domains for mortality and time to viral clearance (appendix p 34). The outcomes of serious adverse events and the duration of admission were limited by not masking participants, health-care providers, data collectors, and outcome assessors (appendix p 34). The plausible worst-case sensitivity analyses indicated that missing data did not statistically significantly influence the observed effects for mortality and serious adverse events (appendix pp 30–31); therefore, we rated the risk of bias for incomplete outcome data as low.

## Discussion

In patients with Ebola virus disease, we found high-certainty evidence that REGN-EB3 or mAb114 reduce mortality compared with ZMapp or remdesivir and moderate-certainty evidence that REGN-EB3 or mAb114 probably reduce mortality relative to standard care. Whether ZMapp or remdesivir reduce mortality compared with standard care is very uncertain. ZMapp might reduce the duration of admission compared with standard care. Compared with standard care, whether REGN-EB3, mAb114, ZMapp, or remdesivir have any important effects on the prevalence of serious adverse events remains uncertain.

Strengths of this systematic review and meta-analysis include the comprehensive search to identify eligible RCTs; independent study selection, data extraction, and risk of bias assessment by two reviewers; network meta-analysis that allowed both direct and indirect comparisons among therapies (REGN-EB3, mAb114, and remdesivir) and standard care that were not directly investigated in RCTs; and application of the GRADE approach to rate the certainty of evidence. To facilitate interpretation of the results, we presented absolute effects. To reflect mortality status in the settings in which clinicians most often administer these drugs, we provided two absolute effect estimates using the lowest and highest mortalities in outbreaks with no fewer than 100 diagnosed patients since 2013.

Our study has some limitations. Due to sparse available data, the results for some comparisons do not have power. The direct comparison of ZMapp versus standard care came from a study evaluating only 71 patients, resulting in very wide CIs and subsequent evidence of very low certainty, which compromised indirect comparisons of the other drugs with standard care. Thus, our inference of evidence of moderate certainty that REGN-EB3 and mAb114 probably reduce mortality compared with standard care relied on indirect comparison and on the high-certainty evidence that REGN-EB3 and mAb114 are superior to ZMapp and remdesivir in the direct comparisons and the low likelihood that ZMapp or remdesivir increase mortality in patients with Ebola virus disease. The two eligible trials^5^, ^6^ included in our study enrolled patients with a diagnosis of Zaire Ebola virus. The effects of therapies could differ among patients with different variations of Ebola virus infection. If other variations of Ebola virus emerge, determining the effect of current antibodies will require further study.

During our outcome selection process, the guideline group members and the survivors of Ebola virus disease were also interested in the risk of onward transmission, functional status after Ebola virus disease, and time to symptom resolution. No trials, however, evaluated therapies for these outcomes. Similarly, subgroup analyses predefined by the guideline panel, including for people who were older than 60 years or pregnant, could not be done due to sparse data. Future trials should ideally address these issues.

The Ct value is an important variable for evaluating disease severity and viral load. The guideline panel recognised this fact and requested a subgroup analysis according to the Ct value. After discussions in a consensus meeting and based on current evidence, the threshold chosen in the eligible trials, and the perception of clinical experts, the panel chose a cutoff value of 22 for the Ct value. In clinical practice, patients with Ebola virus disease and lower Ct values indicating higher viral loads at presentation might die despite effective medical countermeasures.^5^ The two eligible trials^5^, ^6^ dichotomised patients into two groups on the basis of their Ct values (≤22 [corresponding to a higher viral load] or >22 [corresponding to a lower viral load]) and provided evidence consistent with certain interventions being less efficacious in reducing mortality among patients with Ct values equal to or less than versus greater than 22. However, because the data used were from only one trial and conducting multiple subgroup tests increased the risk of a chance-related finding, the subgroup analysis had low credibility; therefore, use of the Ct value in clinical practice requires further investigation.

For the continuous outcomes of time to viral clearance and duration of admission, the two eligible trials did not report data on variability (eg, SD, 95% CI, or IQR) to allow for meta-analysis. We requested data from the authors of the eligible trials; the authors were able to provide some but not other relevant data. We obtained data from the figures in eligible trials for survivors only and used these data to perform analyses. The results for these analyses could deviate from analyses based on all patients. For the outcomes of serious adverse events and duration of admission, due to their open-label design, both trials were rated to be at high risk of bias for the absence of blinding. The certainty of evidence was rated as low or very low for many comparisons due to serious imprecision. As future trials become available for analysis, we anticipate that the certainty rating will improve, and, accordingly, we will periodically update this systematic review and network meta-analysis.

To our knowledge, this systematic review and network meta-analysis is the first to evaluate the efficacy and safety of different therapies for patients with Ebola virus disease. Compared with a previous systematic review^4^ and a previous meta-analysis,^29^ our study focused on evidence from RCTs, used the GRADE approach to assess the certainty of evidence, and presents absolute effects. We included the large PALM trial^6^ that investigated drugs (ie, REGN-EB3, mAb114, and remdesivir) that were not evaluated in previous studies^4^, ^29^ and used indirect comparisons within the network meta-analysis to add additional information to the evidence comparing drugs with standard care. Consistent with previous studies,^4^, ^29^ we found that whether ZMapp reduces mortality compared with standard care is very uncertain. However, our systematic review and meta-analysis establishes the superiority of REGN-EB3 and mAb114 over ZMapp, remdesivir, or standard care with respect to mortality in patients with Ebola virus disease.

There was little or no difference in the prevalence of serious adverse events between REGN-EB3, mAb114, ZMapp, and remdesivir. REGN-EB3 and mAb114 are administered as a single dose, but ZMapp and remdesivir are administered as multiple infusions.^6^ These findings suggest that health-care workers should prioritise the use of REGN-EB3 and mAb114 to treat patients with Ebola virus disease during future outbreaks. However, the effects of the use of monoclonal antibodies in patients with Ebola virus disease on other surrogate outcomes, especially viral mutation and persistence, remain uncertain.

Our review identifies evidence gaps in the treatment of Ebola virus disease and informs further studies (eg, with the creation of a core outcome set). Future trials could focus on additional patient-important outcomes (eg, adverse maternal outcomes, the risk of onward transmission, and functional status after Ebola virus disease), test some clinically important subgroup effects by recruiting more participants to meet required statistical power thresholds (eg, pregnant participants), and include comparisons of new singular therapeutic agents and combination treatments, where appropriate.

This systematic review and network meta-analysis established that REGN-EB3 or mAb114 reduce mortality compared with ZMapp or remdesivir and that REGN-EB3 or mAb114 probably reduce mortality compared with standard care. There was no convincing difference in the prevalence of serious adverse events between REGN-EB3, mAb114, ZMapp, remdesivir, and standard care.

## Data sharing

Data in this systematic review and meta-analysis were extracted from published studies available on the internet. Requests for the meta-analysis data can be made by contacting the corresponding author.

## Declaration of interests

We declare no competing interests.
