# Supplementary material for: Effects of therapies for Ebola virus disease: a systematic review and network meta-analysis
Source: Lancet Microbe. 2022 Sep;3(9):e683–92. doi: 10.1016/S2666-5247(22)00123-9 (PMC9441701; doi:10.1016/S2666-5247(22)00123-9)
Supplement: Supplementary appendix [file mmc1.pdf]

THE LANCET
Microbe

Supplementary appendix

This appendix formed part of the original submission and has been peer reviewed. We post it as supplied by the authors.

Supplement to: Gao Y, Zhao Y, Guyatt G, et al. Effects of therapies for Ebola virus disease: a systematic review and network meta-analysis. *Lancet Microbe* 2022; published online July 5. https://doi.org/10.1016/S2666-5247(22)00123-9.

Effects of therapies for Ebola virus disease: a systematic review and network meta-analysis

Contents

Text S1: Search strategy for each database ... 3

Text S2: Methods for selecting outcomes of interest ... 8

Text S3: Details of data analyses ... 10

Text S4: Details of certainty of evidence rating ... 12

Text S5: Details of risk of bias assessment ... 14

Table S1. Details of standard care ... 15

Tables S2-S4. Direct, indirect, and network treatment estimates for each outcome ... 16

Table S5. Maternal and perinatal outcome data from the PALM Study ... 19

Table S6. GRADE summary of findings for time to viral clearance ... 20

Table S7. GRADE summary of findings for duration of admission ... 21

Tables S8-S11. Subgroup analysis for mortality ... 22

Table S12. Credibility assessment of subgroup analysis for mAb114 versus ZMapp in mortality by cycle-threshold value ... 26

Table S13. Credibility assessment of subgroup analysis for mAb114 versus remdesivir in mortality by cycle-threshold value ... 28

Tables S14-S15. Plausible worst case sensitivity analysis for mortality and serious adverse events ... 30

Figure S1-S2. Network plots ... 32

Figure S3. Risk of bias for each risk of bias item in included studies ... 34

Text S1: Search strategy for each database

Ovid MEDLINE(R) and Epub Ahead of Print, In-Process, In-Data-Review & Other Non-Indexed Citations, Daily and Versions(R) <1946 to Present>

1 hemorrhagic fever, ebola/ or marburg virus disease/

2 (ebola or ebolavirus or EVD or ((Zaire or Bundibugyo or Sudan or Tai Forest or Reston or Bombali) and (ebola or virus))).mp. [mp=title, abstract, original title, name of substance word, subject heading word, floating sub-heading word, keyword heading word, organism supplementary concept word, protocol supplementary concept word, rare disease supplementary concept word, unique identifier, synonyms]

3 1 or 2

4 randomized controlled trial.pt.

5 controlled clinical trial.pt.

6 randomized.ab.

7 placebo.ab.

8 drug therapy.fs.

9 randomly.ab.

10 trial.ti.

11 groups.ab.

12 or/4-11

13 (animals not (humans and animals)).sh.

14 12 not 13

15 3 and 14

Embase <1974 to Present>

1 Ebola hemorrhagic fever/

2 filovirus infection/ or marburg hemorrhagic fever/

3 (ebola or ebolavirus or EVD or ((Zaire or Bundibugyo or Sudan or Tai Forest or Reston or Bombali) and (ebola or virus))).mp. [mp=title, abstract, heading word, drug trade name, original title, device manufacturer, drug manufacturer, device trade name, keyword heading word, floating subheading word, candidate term word]

4 1 or 2 or 3

5 Randomized controlled trial/

6 Controlled clinical study/

7 random$.ti,ab.

8 randomization/

9 intermethod comparison/

10 placebo.ti,ab.

11 (compare or compared or comparison).ti.

12 ((evaluated or evaluate or evaluating or assessed or assess) and (compare or compared or comparing or comparison)).ab.

13 (open adj label).ti,ab.

14 ((double or single or doubly or singly) adj (blind or blinded or blindly)).ti,ab.

15 double blind procedure/

16 parallel group$1.ti,ab.

17 (crossover or cross over).ti,ab.

18 ((assign$ or match or matched or allocation) adj5 (alternate or group$1 or intervention$1 or patient$1 or subject$1 or participant$1)).ti,ab.

19 (assigned or allocated).ti,ab.

20 (controlled adj7 (study or design or trial)).ti,ab.

21 (volunteer or volunteers).ti,ab.

22 human experiment/

23 trial.ti.

24 or/5-23

25 (random$ adj sampl$ adj7 ("cross section$" or questionnaire$1 or survey$ or database$1)).ti,ab. not (comparative study/ or controlled study/ or randomi?ed controlled.ti,ab. or randomly assigned.ti,ab.)

26 Cross-sectional study/ not (randomized controlled trial/ or controlled clinical study/ or controlled study/ or randomi?ed controlled.ti,ab. or control group$1.ti,ab.)

27 (((case adj control$) and random$) not randomi?ed controlled).ti,ab.

28 (Systematic review not (trial or study)).ti.

29 (nonrandom$ not random$).ti,ab.

30 "Random field$".ti,ab.

31 (random cluster adj3 sampl$).ti,ab.

32 (review.ab. and review.pt.) not trial.ti.

33 "we searched".ab. and (review.ti. or review.pt.)

34 "update review".ab.

35 (databases adj4 searched).ab.

36 (rat or rats or mouse or mice or swine or porcine or murine or sheep or lambs or pigs or piglets or rabbit or rabbits or cat or cats or dog or dogs or cattle or bovine or monkey or monkeys or trout or marmoset$1).ti. and animal experiment/
37 Animal experiment/ not (human experiment/ or human/)
38 or/25-37
39 24 not 38
40 4 and 39

Cochrane Central Register of Controlled Trials (CENTRAL)

#1 MeSH descriptor: [Hemorrhagic Fever, Ebola] explode all trees 69
#2 (ebola or ebolavirus or EVD or ((Zaire or Bundibugyo or Sudan or Tai Forest or Reston or Bombali) and (ebola or virus))):ti,ab,kw (Word variations have been searched)
#3 #1 OR #2
Limited Trials

Global Health <1973 to 2021 Week 50>

1 viral haemorrhagic fevers/
2 (ebola or ebolavirus or EVD or ((Zaire or Bundibugyo or Sudan or Tai Forest or Reston or Bombali) and (ebola or virus))).mp. [mp=abstract, title, original title, broad terms, heading words, identifiers, cabicodes]
3 1 or 2
4 randomized controlled trials/
5 (randomized controlled trial or random* or blind* or placebo*).mp. [mp=abstract, title, original title, broad terms, heading words, identifiers, cabicodes]
6 4 or 5
7 3 and 6

Scopus

TITLE-ABS-KEY(ebola or ebolavirus or EVD or ((Zaire or Bundibugyo or Sudan or Tai Forest or Reston or Bombali) and (ebola or virus))) AND TITLE-ABS-KEY("randomized controlled trial" OR random* OR blind* OR placebo*)

CINAHL

S27 S3 AND S26
S26 S25 NOT S24
S25 S4 OR S5 OR S6 OR S7 OR S8 OR S9 OR S10 OR S11 OR S12 OR S13 OR S14 OR S15 OR S16 OR S17 OR S18
S24 S22 NOT S23
S23 MH (human)
S22 S19 OR S20 OR S21
S21 TI (animal model*)
S20 MH (animal studies)
S19 MH animals+
S18 AB (cluster W3 RCT)
S17 MH (crossover design) OR MH (comparative studies)
S16 AB (control W5 group)
S15 PT (randomized controlled trial)
S14 MH (placebos)
S13 MH (sample size) AND AB (assigned OR allocated OR control)
S12 TI (trial)
S11 AB (random*)
S10 TI (randomised OR randomized)
S9 MH cluster sample
S8 MH pretest-posttest design
S7 MH random assignment
S6 MH single-blind studies
S5 MH double-blind studies
S4 MH randomized controlled trials
S3 S1 OR S2
S2 TI (ebola or ebolavirus or EVD or ((Zaire or Bundibugyo or Sudan or Tai Forest or Reston or Bombali) and (ebola or virus))) OR AB (ebola or ebolavirus or EVD or ((Zaire or Bundibugyo or Sudan or Tai Forest or Reston or Bombali) and (ebola or virus)))
S1 MH Ebola hemorrhagic fever

World Health Organization Global Index Medicus (WHOLIS)

Title, abstract, subject: ebola or ebolavirus or EVD or ((Zaire or Bundibugyo or Sudan or Tai Forest or Reston or Bombali) and (ebola or virus))
including:

WPRIM (Western Pacific)
LILACS (Americas)
IMSEAR (South-East Asia)
IMEMR (Eastern Mediterranean)
AIM (Africa)

ClinicalTrials.gov

Condition, disease, other term: ebola or ebolavirus

Epistemonikos

(title:(ebola or ebolavirus or EVD or ((Zaire or Bundibugyo or Sudan or Tai Forest or Reston or Bombali) and (ebola or virus))) OR abstract:(ebola or ebolavirus or EVD or ((Zaire or Bundibugyo or Sudan or Tai Forest or Reston or Bombali) and (ebola or virus)))) AND (title:("randomized controlled trial" OR random* OR blind* OR placebo*) OR abstract:("randomized controlled trial" OR random* OR blind* OR placebo*))

medRxiv

abstract or title "ebola" (match all words)

bioRxiv

abstract or title "ebola" (match all words)

SSRN

abstract or title or keywords "ebola" (match all words)

Text S2: Methods for selecting outcomes of interest

The GDG developed a list of 13 outcomes of interest to patients, families, and healthcare providers. Outcomes were then prioritized through an online survey. The online survey was sent to 38 participants of the WHO steering committee and GDG members. The survey was also sent to five recovered EVD patients in Sierra Leone and five recovered EVD patients in the Democratic Republic of Congo. Participants rated each outcome from 1-9, 7 to 9 - critically important, 4 to 6 – important, 1 to 3 - of limited importance. The survey was provided in both French and English.

25/38 (66%) GDG and WHO steering committee members completed the survey and 10/10 (100%) of EVD patients completed the survey. There were no partial or incomplete responses and no apparent evidence of scale inversion. Survey results were compiled centrally, the results are displayed as mean (SD) in below table:

**Outcome**	**All respondents n=35 mean (SD)**	**GDG participants n=25 mean (SD)**	**EVD survivors n=10 mean (SD)**	**All Rank**	**GDG Rank**	**EVD patients Rank**
Duration of admission	7.3 (1.7)	7.08 (1.7)	7.6 (1.6)	4	4	5
Mortality	8.7 (0.9)	8.8 (0.5)	8.2 (1.4)	1	1	1
Time to symptom resolution	6.8 (1.8)	6.8 (1.9)	6.8 (1.6)	8	7	11
Serious adverse effects	7.1 (1.7)	7.2 (1.3)	7.0 (2.5)	6	3	10
Adverse maternal outcomes	7.5 (1.5)	7.4 (1.2)	7.6 (2.1)	2	2	4
Time to viral clearance	6.5 (2.1)	6.4 (1.9)	6.8 (2.7)	9	9	12
Mental Health outcomes	6.4 (1.8)	6.0 (1.4)	7.2 (2.6)	10	10	8
Adverse perinatal outcomes	6.9 (1.7)	6.8 (1.5)	7.2 (2.4)	7	8	7
Interruption of treatment	5.9 (2.6)	5.8 (2.6)	6 (2.7)	13	12	13
Viraemia through disease course	6.3 (2.7)	5.8 (2.7)	7.5 (2.4)	12	13	6
Functional status post EVD	7.2 (1.6)	6.8 (1.6)	8.1 (1.5)	5	6	3
Risk of onward	7.3 (2.0)	6.9 (1.9)	8.2 (1.9)	3	5	2

transmission						
Future fertility outcomes	6.3 (2.1)	6.0 (1.9)	7.1 (2.3)	11	11	9
Mean outcome prioritization score	6.93 (2.0)	6.78 (1.9)	7.3 (2.2)	-	-	-

Four outcomes were ranked in the top five by both the GDG and EVD patients: mortality, adverse maternal outcomes, duration of admission, risk of onward transmission. The GDG included serious adverse effects in their top five, whilst EVD patients included functional status post EVD. EVD patients reported higher overall mean prioritization scores than GDG members. We included all outcomes with a score ≥ 6.5 as ranked by all participants in our systematic review.

Text S3: Details of data analyses

We performed frequentist network meta-analyses to estimate the effect of all interventions. We planned to use the side-splitting method to evaluate local (loop-specific) incoherence[1,2] in each closed loop of the network as the difference between direct and indirect evidence. However, there was only direct or indirect evidence for each comparison in our analyses, we could not perform the planned analyses.

When data proved available, we performed the following prespecified subgroup analyses:

- Age of patients: ≤ 5 years versus 6 to 59 years versus ≥ 60 years (hypothesis: reduced treatment effect in patients with age ≤ 5 years or ≥ 60 years).
- Prior EVD vaccination: < 10 days versus ≥ 10 days (hypothesis: reduced treatment effect in patients with prior vaccination < 10 days).
- Duration of symptoms prior to treatment: ≤ 5 days versus > 5 days (hypothesis: reduced treatment effect in patients with symptoms > 5 days).
- Pregnancy: pregnant versus non-pregnant (hypothesis: reduced treatment effect in pregnant patients).
- Cycle-threshold (Ct) value (a value used to measure Ebola virus RNA levels): Ct ≤ 22 versus Ct > 22 (hypothesis: reduced treatment effect in patients with Ct ≤ 22).

Data proved available to perform subgroup analyses by age, Ct value, EVD vaccination status (PALM trial authors provided data by self-report EVD vaccine status: vaccinated and unvaccinated), and duration of symptoms (illness) (PALM trial authors provided data with a cutoff of 4 days, which is also the median of all participants) at baseline for mortality. Using within-trial information, we first performed frequentist network meta-analyses for each subgroup separately. Then we used network estimates of each subgroup to calculate the P interaction across three age groups (age ≤ 5 years, 6 to 17 years, and ≥ 18 years), and between Ct > 22 and Ct ≤ 22, self-report vaccinated and unvaccinated patients, and between baseline symptoms ≤ 4 days and > 4 days for each comparison. We assessed the credibility of possible subgroup analysis using the Instrument for assessing the Credibility of Effect

Modification Analyses (ICEMAN) tool.[3]

Reference

1. Higgins JP, Thompson SG, Deeks JJ, Altman DG. Measuring inconsistency in meta-analyses. BMJ 2003; 327(7414): 557-60.
2. Lu G, Ades AE. Assessing Evidence Inconsistency in Mixed Treatment Comparisons. Journal of the American Statistical Association 2006; 101(474): 447-59.
3. Schandelmaier S, Briel M, Varadhan R, et al. Development of the Instrument to assess the Credibility of Effect Modification Analyses (ICEMAN) in randomized controlled trials and meta-analyses. CMAJ 2020; 192(32): E901-06.

Text S4: Details of certainty of evidence rating

We rated the certainty of evidence for direct comparison by assessing domains of the risk of bias, inconsistency, indirectness, imprecision, and publication bias.[1-8] To assess the impact of missing outcome data, we performed a plausible worst case sensitivity analysis for each outcome.[9] Whenever missing data imputation strategies did not significantly influence the observed effect, we rated the assessment of risk of bias for missing information as “definitely low risk of bias” for all trials.

Certainty ratings of indirect estimates started at the lowest rating of the direct comparisons that contribute to the most-dominant first order loop except for uncertainty only due to imprecision. We further rated down for intransitivity only if there was evidence of plausible effect modification between the direct comparisons that inform the indirect comparison. We assessed the transitivity assumption underlying network meta-analysis comparing the distribution of population, intervention, and methodological characteristics of studies across treatment comparisons.

For the certainty of network estimates, we started with the estimate - direct or indirect - that dominates the network estimate. We assessed imprecision at the network level by comparing the confidence intervals to thresholds[4] agreed by the guideline panel for each outcome. We used the MID threshold for mortality as 1% and serious adverse events as 2%; for time to viral clearance and duration of admission at 1 day. We rated down one level for imprecision if the 95% CI crossed either side of the MID threshold; we rated down two levels for imprecision if the 95% CI crossed both sides of the MID threshold; we rated down three levels for imprecision if the 95% CI included both a large benefit and a large harm.

If incoherence was present, we rated down the certainty of the network estimates and used, as the best estimate, that with the higher certainty of the direct and indirect evidence. We developed a summary of findings table for each paired comparison for each outcome. We generated two rows for mortality for each

comparison: one row presents absolute risk estimating from the lowest baseline risk, the other row presents absolute risk estimating from the highest baseline risk.

Reference

1. Guyatt GH, Oxman AD, Vist GE, et al. GRADE: an emerging consensus on rating quality of evidence and strength of recommendations. BMJ 2008; 336(7650): 924-6.
2. Guyatt GH, Oxman AD, Vist G, et al. GRADE guidelines: 4. Rating the quality of evidence--study limitations (risk of bias). J Clin Epidemiol 2011; 64(4): 407-15.
3. Guyatt GH, Oxman AD, Montori V, et al. GRADE guidelines: 5. Rating the quality of evidence--publication bias. J Clin Epidemiol 2011; 64(12): 1277-82.
4. Guyatt GH, Oxman AD, Kunz R, et al. GRADE guidelines 6. Rating the quality of evidence--imprecision. J Clin Epidemiol 2011; 64(12): 1283-93.
5. Guyatt GH, Oxman AD, Kunz R, et al. GRADE guidelines: 7. Rating the quality of evidence--inconsistency. J Clin Epidemiol 2011; 64(12): 1294-302.
6. Guyatt GH, Oxman AD, Kunz R, et al. GRADE guidelines: 8. Rating the quality of evidence--indirectness. J Clin Epidemiol 2011; 64(12): 1303-10.
7. Brignardello-Petersen R, Murad MH, Walter SD, et al. GRADE approach to rate the certainty from a network meta-analysis: avoiding spurious judgments of imprecision in sparse networks. J Clin Epidemiol 2019; 105: 60-7.
8. Brignardello-Petersen R, Mustafa RA, Siemieniuk RAC, et al. GRADE approach to rate the certainty from a network meta-analysis: addressing incoherence. J Clin Epidemiol 2019; 108: 77-85.
9. Guyatt GH, Ebrahim S, Alonso-Coello P, et al. GRADE guidelines 17: assessing the risk of bias associated with missing participant outcome data in a body of evidence. J Clin Epidemiol 2017; 87: 14-22.

Text S5: Details of risk of bias assessment

Two reviewers (YG and YZ) assessed the risk of bias of included randomized controlled trials using a modified Cochrane risk of bias tool.[1] Reviewers resolved disagreement by discussion and, when necessary, with adjudication by a third reviewer. The instrument includes the following domains: random sequence generation; allocation concealment; blinding of participants, healthcare providers, data collectors, outcome assessor/adjudicator, data analysts; incomplete outcome data; and other sources of bias (e.g. baseline imbalance). We rated each domain at the outcome level as either: definitely or probably low risk of bias (low risk of bias), probably or definitely high risk of bias (high risk of bias).

Reference

1. Guyatt GH, Busse JW. Modification of cochrane tool to assess risk of bias in randomized trials. ttps://www.evidencepartners.com/resources/methodological-resources/. Accessed November 30, 2021.

Table S1. Details of standard care

Study	Details of standard care
Davey (2016), PREVAIL II	Hemodynamic monitoring, the provision of intravenous fluids, laboratory testing, and delivery of concomitant medications
Mulangu (2019), PALM	Administration of intravenous fluids, daily clinical laboratory testing, correction of hypoglycemia and electrolyte imbalances, and administration of broad-spectrum antibiotic agents and antimalarial agents as indicated

Tables S2-S4. Direct, indirect, and network treatment estimates for each outcome

Table S2. Direct, indirect, and network treatment estimates for mortality

Comparison	K	Direct estimate	Indirect estimate	Network estimate
REGN-EB3 versus standard care	0	NA	0.40 (0.18, 0.89)	0.40 (0.18, 0.89)
mAb114 versus standard care	0	NA	0.42 (0.19, 0.93)	0.42 (0.19, 0.93)
ZMapp versus standard care	1	0.60 (0.28, 1.26)	NA	0.60 (0.28, 1.26)
Remdesivir versus standard care	0	NA	0.64 (0.29, 1.39)	0.64 (0.29, 1.39)
REGN-EB3 versus mAb114	1	0.96 (0.71, 1.29)	NA	0.96 (0.71, 1.29)
REGN-EB3 versus ZMapp	1	0.67 (0.52, 0.88)	NA	0.67 (0.52, 0.88)
REGN-EB3 versus remdesivir	1	0.63 (0.49, 0.82)	NA	0.63 (0.49, 0.82)
mAb114 versus ZMapp	1	0.71 (0.55, 0.91)	NA	0.71 (0.55, 0.91)
mAb114 versus remdesivir	1	0.66 (0.52, 0.84)	NA	0.66 (0.52, 0.84)
ZMapp versus remdesivir	1	0.94 (0.76, 1.15)	NA	0.94 (0.76, 1.15)

Values are relative risk (95% CI). Comparison: Treatment comparison, K: Number of studies providing direct evidence.

Table S3. Direct, indirect, and network treatment estimates for serious adverse events

Comparison	K	Direct estimate	Indirect estimate	Network estimate
REGN-EB3 versus standard care	0	NA	0.016 (-0.061, 0.093)	0.016 (-0.061, 0.093)
mAb114 versus standard care	0	NA	0.016 (-0.061, 0.093)	0.016 (-0.061, 0.093)
ZMapp versus standard care	1	0.028 (-0.046, 0.102)	NA	0.028 (-0.046, 0.102)
Remdesivir versus standard care	0	NA	0.022 (-0.056, 0.099)	0.022 (-0.056, 0.099)
REGN-EB3 versus mAb114	1	0.000 (-0.012, 0.012)	NA	0.000 (-0.012, 0.012)
REGN-EB3 versus ZMapp	1	-0.012 (-0.032, 0.008)	NA	-0.012 (-0.032, 0.008)
REGN-EB3 versus remdesivir	1	-0.006 (-0.022, 0.011)	NA	-0.006 (-0.022, 0.011)
mAb114 versus ZMapp	1	-0.012 (-0.032, 0.008)	NA	-0.012 (-0.032, 0.008)
mAb114 versus remdesivir	1	-0.006 (-0.021, 0.010)	NA	-0.006 (-0.021, 0.010)
ZMapp versus remdesivir	1	0.006 (-0.017, 0.029)	NA	0.006 (-0.017, 0.029)

Values are risk difference (95% CI). Comparison: Treatment comparison, K: Number of studies providing direct evidence.

Table S4. Direct, indirect, and network treatment estimates for time to viral clearance

Comparison	K	Direct estimate	Indirect estimate	Network estimate
REGN-EB3 versus standard care	0	NA	-0.30 (-3.20, 2.60)	-0.30 (-3.20, 2.60)
mAb114 versus standard care	0	NA	-1.14 (-4.09, 1.81)	-1.14 (-4.09, 1.81)
ZMapp versus standard care	1	-0.25 (-2.70, 2.20)	NA	-0.25 (-2.70, 2.20)
Remdesivir versus standard care	0	NA	-0.27 (-3.23, 2.69)	-0.27 (-3.23, 2.69)
REGN-EB3 versus mAb114	1	0.84 (-0.68, 2.36)	NA	0.84 (-0.68, 2.36)
REGN-EB3 versus ZMapp	1	-0.05 (-1.60, 1.50)	NA	-0.05 (-1.60, 1.50)
REGN-EB3 versus remdesivir	1	-0.03 (-1.56, 1.50)	NA	-0.03 (-1.56, 1.50)
mAb114 versus ZMapp	1	-0.89 (-2.54, 0.76)	NA	-0.89 (-2.54, 0.76)
mAb114 versus remdesivir	1	-0.87 (-2.50, 0.76)	NA	-0.87 (-2.50, 0.76)
ZMapp versus remdesivir	1	0.02 (-1.63, 1.67)	NA	0.02 (-1.63, 1.67)

Values are mean difference (95% CI). Comparison: Treatment comparison, K: Number of studies providing direct evidence.

Table S5. Maternal and perinatal outcome data from the PALM Study

Outcomes	Overall	ZMapp	Remdesivir	mAb114	REGN-EB3
Number of pregnancies reported	18	4	7	5	2
Number with pregnancy outcome data available[a]	17	4	6	5	2
Number of maternal deaths by 28 days	9	1	5	2	1
Number of embryo-fetal losses	14	3	5	5	1
Due to maternal death	9	1	5	2	1
Due to other complications[b]	5	2	0	3	0
Number of live births	3	1	1	0	1

[a] One pregnant participant on the remdesivir arm of the trial completed the final study visit at 58 days but was later unable to be located to determine the pregnancy outcome.
[b] A pharmacovigilance working group assessment indicated that all embryo-fetal deaths for reasons other than maternal death were related to the impact of maternal EVD on the fetus and could not be attributed to study product received.

Table S6. GRADE summary of findings for time to viral clearance

Comparison	Mean difference (95% CI)	Certainty in effect estimates	Plain language summary
REGN-EB3 versus standard care	-0.30 (-3.20 to 2.60)	Low‡	REGN-EB3 might have little or no effect on time to viral clearance compared with standard care.
mAb114 versus standard care	-1.14 (-4.09 to 1.81)	Low‡	mAb114 might have little or no effect on time to viral clearance compared with standard care.
ZMapp versus standard care	-0.25 (-2.70 to 2.20)	Low‡	ZMapp might have little or no effect on time to viral clearance compared with standard care.
Remdesivir versus standard care	-0.27 (-3.23 to 2.69)	Low‡	Remdesivir might have little or no effect on time to viral clearance compared with standard care.
REGN-EB3 versus mAb114	0.84 (-0.68 to 2.36)	Moderate†	REGN-EB3 probably has little or no effect on time to viral clearance compared with mAb114.
REGN-EB3 versus ZMapp	-0.05 (-1.60 to 1.50)	Low‡	There might be little or no difference between REGN-EB3 and ZMapp on time to viral clearance.
REGN-EB3 versus remdesivir	-0.03 (-1.56 to 1.50)	Low‡	There might be little or no difference between REGN-EB3 and remdesivir on time to viral clearance.
mAb114 versus ZMapp	-0.89 (-2.54 to 0.76)	Moderate†	mAb114 probably has little or no effect on time to viral clearance compared with ZMapp.
mAb114 versus remdesivir	-0.87 (-2.50 to 0.76)	Moderate†	mAb114 probably has little or no effect on time to viral clearance compared with remdesivir.
ZMapp versus remdesivir	0.02 (-1.63 to 1.67)	Low‡	There might be little or no difference between ZMapp and remdesivir on time to viral clearance.

†Rated down for imprecision.

‡Rated down 2 levels for imprecision.

Table S7. GRADE summary of findings for duration of admission

Comparison	Mean difference (95% CI)	Certainty in effect estimates	Plain language summary
ZMapp versus standard care	-2.02 (-4.05 to 0.01)	Low*†	ZMapp might reduce the duration of admission compared with standard care.

*Rated down for risk of bias.

†Rated down for imprecision.

Tables S8-S11. Subgroup analysis for mortality

Table S8. Subgroup analysis for mortality by age grouping

Comparison	Age≤5	Age 6 to 17	Age≥18	Subgroup difference (P value)
REGN-EB3 versus standard care	0.29 (0.04 to 1.90)	0.79 (0.04 to 15.59)	0.49 (0.18 to 1.30)	0.835
mAb114 versus standard care	0.29 (0.05 to 1.91)	1.10 (0.06 to 20.16)	0.50 (0.19 to 1.33)	0.736
ZMapp versus standard care	0.31 (0.05 to 1.80)	1.52 (0.09 to 24.79)	0.74 (0.29 to 1.89)	0.583
Remdesivir versus standard care	0.43 (0.07 to 2.78)	1.70 (0.10 to 30.50)	0.77 (0.29 to 2.01)	0.717
REGN-EB3 versus mAb114	0.98 (0.51 to 1.89)	0.72 (0.26 to 1.99)	0.98 (0.68 to 1.40)	0.851
REGN-EB3 versus ZMapp	0.93 (0.47 to 1.82)	0.52 (0.18 to 1.49)	0.66 (0.48 to 0.89)	0.577
REGN-EB3 versus remdesivir	0.67 (0.36 to 1.22)	0.46 (0.18 to 1.20)	0.63 (0.47 to 0.86)	0.798
mAb114 versus ZMapp	0.94 (0.49 to 1.82)	0.72 (0.32 to 1.63)	0.67 (0.50 to 0.91)	0.654
mAb114 versus remdesivir	0.68 (0.38 to 1.22)	0.65 (0.33 to 1.27)	0.65 (0.48 to 0.87)	0.991
ZMapp versus remdesivir	0.72 (0.39 to 1.33)	0.89 (0.43 to 1.85)	0.96 (0.77 to 1.21)	0.685

Note: The pre-defined subgroup by age: ≤ 5 years versus 6 to 59 years versus ≥ 60 years. Values are relative risk (95% CI). All subgroup information was from only 1 trial.

Table S9. Subgroup analysis for mortality by cycle-threshold value

Comparison	Ct>22	Ct≤22	Subgroup difference (P value)
REGN-EB3 versus standard care	0.11 (0.01 to 1.00)	0.59 (0.29 to 1.19)	0.172
mAb114 versus standard care	0.10 (0.01 to 0.88)	0.64 (0.32 to 1.30)	0.121
ZMapp versus standard care	0.24 (0.03 to 1.95)	0.78 (0.39 to 1.54)	0.293
Remdesivir versus standard care	0.28 (0.03 to 2.43)	0.79 (0.39 to 1.57)	0.378
REGN-EB3 versus mAb114	1.13 (0.50 to 2.60)	0.91 (0.72 to 1.15)	0.620
REGN-EB3 versus ZMapp	0.46 (0.23 to 0.91)	0.75 (0.61 to 0.93)	0.183
REGN-EB3 versus remdesivir	0.39 (0.20 to 0.75)	0.75 (0.61 to 0.92)	0.064
mAb114 versus ZMapp	0.40 (0.20 to 0.80)	0.83 (0.69 to 0.99)	0.046
mAb114 versus remdesivir	0.34 (0.18 to 0.66)	0.82 (0.69 to 0.98)	0.010
ZMapp versus remdesivir	0.84 (0.53 to 1.34)	0.99 (0.86 to 1.14)	0.506

Values are relative risk (95% CI). Ct: cycle-threshold value, a value used to measure Ebola virus RNA levels. All subgroup information was from only 1 trial.

Table 10. Subgroup analysis for mortality by prior Ebola virus disease vaccination

Comparison	Prior vaccinated	Prior unvaccinated	Subgroup difference (P value)
REGN-EB3 vs. mAb114	0.70 (0.43 to 1.12)	0.94 (0.76 to 1.16)	0.269
REGN-EB3 vs. ZMapp	0.50 (0.29 to 0.86)	0.78 (0.62 to 0.97)	0.138
REGN-EB3 vs. remdesivir	0.52 (0.30 to 0.91)	0.76 (0.61 to 0.94)	0.212
mAb114 vs. ZMapp	0.72 (0.43 to 1.18)	0.83 (0.67 to 1.03)	0.612
mAb114 vs. remdesivir	0.75 (0.45 to 1.24)	0.81 (0.65 to 0.99)	0.783
ZMapp vs. remdesivir	1.05 (0.59 to 1.86)	0.97 (0.78 to 1.22)	0.801

Note: The pre-defined subgroup by prior EVD vaccination: < 10 days versus ≥ 10 days. Values are relative risk (95% CI). All subgroup information was from PALM trial. Data from both the 4-arm main phase and the 2-arm (mAb114 vs REGN-EB3) extension phase of the trial (The trial authors provided requested data). Vaccination data is participant self-reported.

Table 11. Subgroup analysis for mortality by duration of symptoms at baseline

Comparison	Symptoms≤4	Symptoms>4	Subgroup difference (P value)
REGN-EB3 vs. mAb114	1.06 (0.77 to 1.45)	0.82 (0.65 to 1.04)	0.202
REGN-EB3 vs. ZMapp	0.66 (0.47 to 0.93)	0.79 (0.61 to 1.03)	0.413
REGN-EB3 vs. remdesivir	0.65 (0.47 to 0.90)	0.72 (0.56 to 0.92)	0.624
mAb114 vs. ZMapp	0.63 (0.44 to 0.89)	0.96 (0.76 to 1.21)	0.050
mAb114 vs. remdesivir	0.62 (0.44 to 0.86)	0.87 (0.71 to 1.08)	0.093
ZMapp vs. remdesivir	0.98 (0.69 to 1.40)	0.91 (0.71 to 1.16)	0.736

Note: The pre-defined subgroup by duration of symptoms at baseline: ≤ 5 days versus > 5 days. Values are relative risk (95% CI). All subgroup information was from PALM trial. Data from both the 4-arm main phase and the 2-arm (mAb114 vs REGN-EB3) extension phase of the trial (The trial authors provided requested data).

Table S12. Credibility assessment of subgroup analysis for mAb114 versus ZMapp in mortality by cycle-threshold value

Credibility assessment

1: Is the analysis of effect modification based on comparison within rather than between trials?

[ ] Completely between	[ ] Mostly between or unclear	[ ] Mostly within	[X] Completely within
Subgroup analysis or meta-regression comparing overall effects of each individual trial. This is typical for aggregate data meta-analysis.	*Subgroup analysis or meta-regression with most information coming from overall effects, but some trials providing within-trial subgroup information*	*Most trials providing within-trial subgroup information; or individual participant data analysis that combines within and between trial information*	*All trials providing within-trial subgroup information or individual participant data; and the analysis separates within from between trial information, e.g., meta-analysis of interactions*

Comment: 1 trial provided within subgroups used for analysis.

2: For within-trial comparisons, is the effect modification similar from trial to trial? [**X**] Not applicable: no or one within-RCT comparison

[ ] Definitely not similar	[ ] Probably not similar or unclear	[ ] Mostly similar	[ ] Definitely similar
Effect modification reported for two or more trials and clearly different directions	*Effect modification not reported for individual trials or too imprecise to tell*	*Effect modification reported for two or more trials, mostly similar in direction, but considerable differences in magnitude*	*Effect modification reported for two or more trials, similar in direction, only some differences in magnitude*

Comment: 1 trial provided within subgroups used for analysis.

3: For between-trial comparisons, is the number of trials large? [**X**] Not applicable: no between RCT comparison

[ ] Very small	[ ] Rather small or unclear	[ ] Rather large	[ ] Large
1 or 2 or in smallest subgroup; 5 or less in continuous meta-regression	*3-4 in smallest subgroup; 6-10 in continuous meta-regression*	*5-9 in smallest subgroup; 11 to 15 in continuous meta-regression*	*10 or more in smallest subgroup; more than 15 in continuous meta-regression*

Comment:

4: Was the direction of effect modification correctly hypothesized a priori?

[ ] Definitely no	[ ] Probably no or unclear	[ ] Probably yes	[X] Definitely yes
Clearly post-hoc or results inconsistent with hypothesized direction or biologically very implausible	*Vague hypothesis or hypothesized direction unclear*	*No prior protocol available but unequivocal statement of a priori hypothesis with correct direction of effect modification*	*Prior protocol available and includes correct specification of direction of effect modification, e.g., based on a biologic rationale*

Comment: Reduced treatment effect in patients with Ct≤22.

5: Does a test for interaction suggest that chance is an unlikely explanation of the apparent effect modification? (consider irrespective of number of effect modifiers)

[ ] Chance a very likely explanation	[X] Chance a likely explanation or unclear	[ ] Chance may not explain	[ ] Chance an unlikely explanation
Interaction or meta-regression p-value >0.05	*Interaction or meta-regression p-value ≤0.05 and >0.01, or no test of interaction reported and not computable*	*Interaction or meta-regression p-value ≤0.01 and >0.005*	*Interaction or meta-regression p-value ≤0.005*

Comment: P=0.046

6: Did the authors test only a small number of effect modifiers or consider the number in their statistical analysis?

[ ] Definitely no	[X] Probably no or unclear	[ ] Probably yes	[ ] Definitely yes
Explicitly exploratory analysis or large number of effect modifiers tested (e.g., greater than 10) and multiplicity not considered in analysis	*No mention of number or 4-10 effect modifiers tested and number not considered in analysis*	*No protocol available but unequivocal statement of 3 or fewer effect modifiers tested*	*Protocol available and 3 or fewer effect modifiers tested or number considered in analysis*

Comment: Tested 2 effect modifiers for 10 comparisons.

7: Did the authors use a random effects model? [**X**] Not applicable

[ ] Definitely no	[ ] Probably no or unclear	[ ] Probably yes	[ ] Definitely yes
Fixed (or common) effect or fixed effects model explicitly stated	*Probably fixed effect(s) model*	*Probably random (or mixed) effects*	*Random (or mixed) effects explicitly stated*

Comment: No data synthesis.

8: If the effect modifier is a continuous variable, were arbitrary cut points avoided? [**X**] not applicable: not continuous

[ ] Definitely no	[ ] Probably no or unclear	[ ] Probably yes	[ ] Definitely yes
Analysis based on exploratory cut point(s), e.g., picking cut point associated with highest interaction p-value	*Analysis based on cut point(s) of unclear origin*	*Analysis based on pre-specified cut point(s), e.g., suggested by prior RCT*	*Analysis based on the full continuum, e.g., assuming a linear or logarithmic relationship*

Comment:

9 Optional: Are there any additional considerations that may increase or decrease credibility? (manual section 3.9) [ ] not applicable

	[ ] Yes, probably decrease	[ ] Yes, probably increase	
	Biologically implausible<br>Expect similar severe critical<br>Opposite effects unlikely		

Comment: The cut point for categorization appears to be data driven

The number of events driving the p-value is extremely small

Biology seems very dubious

10: How would you rate the overall credibility of the proposed effect modification?
The overall rating should be driven by the items that decrease credibility. The following provides a sensible strategy:

- All responses definitely or probably decrease credibility or unclear → very low
- Two or more responses definitely decrease credibility → maximum usually low even if all other responses satisfy credibility criteria
- One response definitely decreases credibility → maximum usually moderate even if all other responses satisfy credibility criteria
- Two responses probably decrease credibility → maximum usually moderate even if all other responses satisfy credibility criteria
- No response options definitely or probably decrease credibility → high very likely

Place a mark on the continuous line (or type "x" in editable version)

X

**Very low credibility**	**Low credibility**	**Moderate credibility**	**High credibility**
Very likely no effect modification<br>Use overall effect for each subgroup	Likely no effect modification<br>Use overall effect for each subgroup but note remaining uncertainty	Likely effect modification<br>Use separate effects for each subgroup but note remaining uncertainty	Very likely effect modification<br>Use separate effects for each subgroup

Comment: Chance remains a likely explanation for the finding and we conducted multiple subgroup tests.

Table S13. Credibility assessment of subgroup analysis for mAb114 versus remdesivir in mortality by cycle-threshold value

Credibility assessment

1: Is the analysis of effect modification based on comparison within rather than between trials?

[ ] Completely between	[ ] Mostly between or unclear	[ ] Mostly within	[X] Completely within
Subgroup analysis or meta-regression comparing overall effects of each individual trial. This is typical for aggregate data meta-analysis.	*Subgroup analysis or meta-regression with most information coming from overall effects, but some trials providing within-trial subgroup information*	*Most trials providing within-trial subgroup information; or individual participant data analysis that combines within and between trial information*	*All trials providing within-trial subgroup information or individual participant data; and the analysis separates within from between trial information, e.g., meta-analysis of interactions*

Comment: 1 trial provided within subgroups used for analysis.

2: For within-trial comparisons, is the effect modification similar from trial to trial? [X] Not applicable: no or one within-RCT comparison

[ ] Definitely not similar	[ ] Probably not similar or unclear	[ ] Mostly similar	[ ] Definitely similar
Effect modification reported for two or more trials and clearly different directions	*Effect modification not reported for individual trials or too imprecise to tell*	*Effect modification reported for two or more trials, mostly similar in direction, but considerable differences in magnitude*	*Effect modification reported for two or more trials, similar in direction, only some differences in magnitude*

Comment: 1 trial provided within subgroups used for analysis.

3: For between-trial comparisons, is the number of trials large? [X] Not applicable: no between RCT comparison

[ ] Very small	[ ] Rather small or unclear	[ ] Rather large	[ ] Large
1 or 2 or in smallest subgroup; 5 or less in continuous meta-regression	*3-4 in smallest subgroup; 6-10 in continuous meta-regression*	*5-9 in smallest subgroup; 11 to 15 in continuous meta-regression*	*10 or more in smallest subgroup; more than 15 in continuous meta-regression*

Comment:

4: Was the direction of effect modification correctly hypothesized a priori?

[ ] Definitely no	[ ] Probably no or unclear	[ ] Probably yes	[X] Definitely yes
Clearly post-hoc or results inconsistent with hypothesized direction or biologically very implausible	*Vague hypothesis or hypothesized direction unclear*	*No prior protocol available but unequivocal statement of a priori hypothesis with correct direction of effect modification*	*Prior protocol available and includes correct specification of direction of effect modification, e.g., based on a biologic rationale*

Comment: Reduced treatment effect in patients with Ct≤22.

5: Does a test for interaction suggest that chance is an unlikely explanation of the apparent effect modification? (consider irrespective of number of effect modifiers)

[ ] Chance a very likely explanation	[ ] Chance a likely explanation or unclear	[X] Chance may not explain	[ ] Chance an unlikely explanation
Interaction or meta-regression p-value >0.05	*Interaction or meta-regression p-value ≤0.05 and >0.01, or no test of interaction reported and not computable*	*Interaction or meta-regression p-value ≤0.01 and >0.005*	*Interaction or meta-regression p-value ≤0.005*

Comment: P=0.01

6: Did the authors test only a small number of effect modifiers or consider the number in their statistical analysis?

[ ] Definitely no	[X] Probably no or unclear	[ ] Probably yes	[ ] Definitely yes
Explicitly exploratory analysis or large number of effect modifiers tested (e.g., greater than 10) and multiplicity not considered in analysis	*No mention of number or 4-10 effect modifiers tested and number not considered in analysis*	*No protocol available but unequivocal statement of 3 or fewer effect modifiers tested*	*Protocol available and 3 or fewer effect modifiers tested or number considered in analysis*

Comment: Tested 2 effect modifiers for 10 comparisons.

7: Did the authors use a random effects model? [X] Not applicable

[ ] Definitely no	[ ] Probably no or unclear	[ ] Probably yes	[ ] Definitely yes
Fixed (or common) effect or fixed effects model explicitly stated	*Probably fixed effect(s) model*	*Probably random (or mixed) effects*	*Random (or mixed) effects explicitly stated*

Comment: No data synthesis.

8: If the effect modifier is a continuous variable, were arbitrary cut points avoided? [**X**] not applicable: not continuous

[ ] Definitely no	[ ] Probably no or unclear	[ ] Probably yes	[ ] Definitely yes
Analysis based on exploratory cut point(s), e.g., picking cut point associated with highest interaction p-value	*Analysis based on cut point(s) of unclear origin*	*Analysis based on pre-specified cut point(s), e.g., suggested by prior RCT*	*Analysis based on the full continuum, e.g., assuming a linear or logarithmic relationship*

Comment:

---

9 Optional: Are there any additional considerations that may increase or decrease credibility? (manual section 3.9) [ ] not applicable

[ ] Yes, probably decrease<br>Biologically implausible<br>Expect similar severe critical<br>Opposite effects unlikely	[ ] Yes, probably increase

Comment: The cut point for categorization appears to be data driven

The number of events driving the p-value is extremely small

Biology seems very dubious

10: How would you rate the overall credibility of the proposed effect modification?

The overall rating should be driven by the items that decrease credibility. The following provides a sensible strategy:

- All responses definitely or probably decrease credibility or unclear → very low
- Two or more responses definitely decrease credibility → maximum usually low even if all other responses satisfy credibility criteria
- One response definitely decreases credibility → maximum usually moderate even if all other responses satisfy credibility criteria
- Two responses probably decrease credibility → maximum usually moderate even if all other responses satisfy credibility criteria
- No response options definitely or probably decrease credibility → high very likely

Place a mark on the continuous line (or type "x" in editable version)

X

**Very low credibility**	**Low credibility**	**Moderate credibility**	**High credibility**
Very likely no effect modification<br>Use overall effect for each subgroup	Likely no effect modification<br>Use overall effect for each subgroup but note remaining uncertainty	Likely effect modification<br>Use separate effects for each subgroup but note remaining uncertainty	Very likely effect modification<br>Use separate effects for each subgroup

Comment: Chance remains a likely explanation for the finding and we conducted multiple subgroup tests.

Tables S14-S15. Plausible worst case sensitivity analysis for mortality and serious adverse events

Table S14. Plausible worst case sensitivity analysis for mortality ($RI_{LTFU/FU}$ for intervention group=5, $RI_{LTFU/FU}$ for control group=1)

Comparison	K	Direct estimate	Indirect estimate	Network estimate
REGN-EB3 versus standard care	0	NA	0.43 (0.19, 0.95)	0.43 (0.19, 0.95)
mAb114 versus standard care	0	NA	0.44 (0.20, 0.98)	0.44 (0.20, 0.98)
ZMapp versus standard care	1	0.62 (0.29, 1.30)	NA	0.62 (0.29, 1.30)
Remdesivir versus standard care	0	NA	0.66 (0.31, 1.45)	0.66 (0.31, 1.45)
REGN-EB3 versus mAb114	1	0.97 (0.73, 1.30)	NA	0.97 (0.73, 1.30)
REGN-EB3 versus ZMapp	1	0.70 (0.54, 0.91)	NA	0.70 (0.54, 0.91)
REGN-EB3 versus remdesivir	1	0.65 (0.50, 0.84)	NA	0.65 (0.50, 0.84)
mAb114 versus ZMapp	1	0.72 (0.56, 0.92)	NA	0.72 (0.56, 0.92)
mAb114 versus remdesivir	1	0.67 (0.52, 0.85)	NA	0.67 (0.52, 0.85)
ZMapp versus remdesivir	1	0.93 (0.75, 1.14)	NA	0.93 (0.75, 1.14)

Values are relative risk (95% CI). Comparison: Treatment comparison, K: Number of studies providing direct evidence. NA: Not applicable.

Table S15. Plausible worst case sensitivity analysis for serious adverse events ($RI_{LTFU/FU}$ for intervention group=5, $RI_{LTFU/FU}$ for control group=1)

Comparison	K	Direct estimate	Indirect estimate	Network estimate
REGN-EB3 versus standard care	0	NA	0.016 (-0.060, 0.092)	0.016 (-0.060, 0.092)
mAb114 versus standard care	0	NA	0.016 (-0.060, 0.092)	0.016 (-0.060, 0.092)
ZMapp versus standard care	1	0.028 (-0.046, 0.101)	NA	0.028 (-0.046, 0.101)
Remdesivir versus standard care	0	NA	0.022 (-0.055, 0.099)	0.022 (-0.055, 0.099)
REGN-EB3 versus mAb114	1	0.000 (-0.012, 0.012)	NA	0.000 (-0.012, 0.012)
REGN-EB3 versus ZMapp	1	-0.012 (-0.032, 0.008)	NA	-0.012 (-0.032, 0.008)
REGN-EB3 versus remdesivir	1	-0.006 (-0.022, 0.010)	NA	-0.006 (-0.022, 0.010)
mAb114 versus ZMapp	1	-0.012 (-0.032, 0.008)	NA	-0.012 (-0.032, 0.008)
mAb114 versus remdesivir	1	-0.006 (-0.021, 0.010)	NA	-0.006 (-0.021, 0.010)
ZMapp versus remdesivir	1	0.006 (-0.016, 0.029)	NA	0.006 (-0.016, 0.029)

Values are risk difference (95% CI). Comparison: Treatment comparison, K: Number of studies providing direct evidence. NA: Not applicable.

Figure S1-S2. Network plots

*The size of the circle represents the number of participants. The connecting lines represent direct comparisons. The width of the line represents the number of studies.

Figure S1. Network plot for serious adverse events

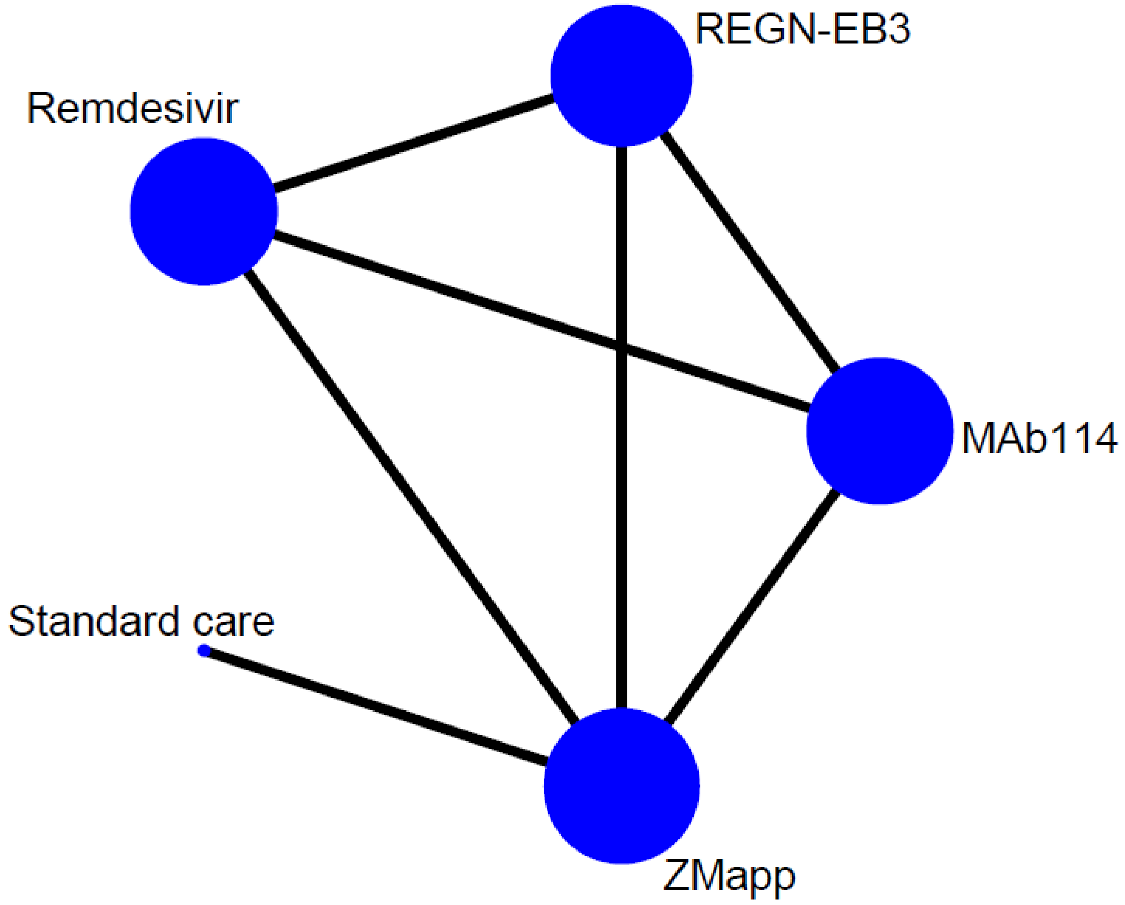

Figure S2. Network plot for time to viral clearance

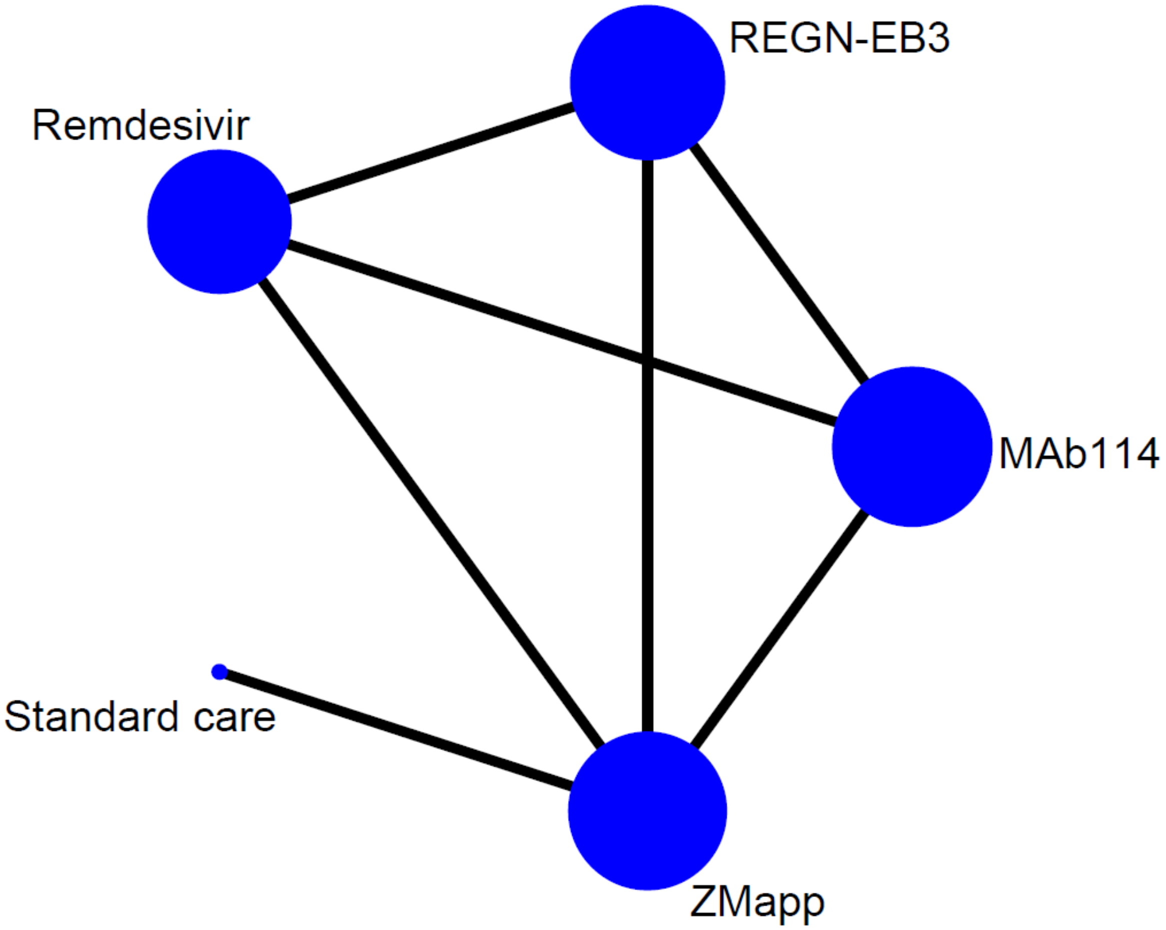

Figure S3. Risk of bias for each risk of bias item in included studies

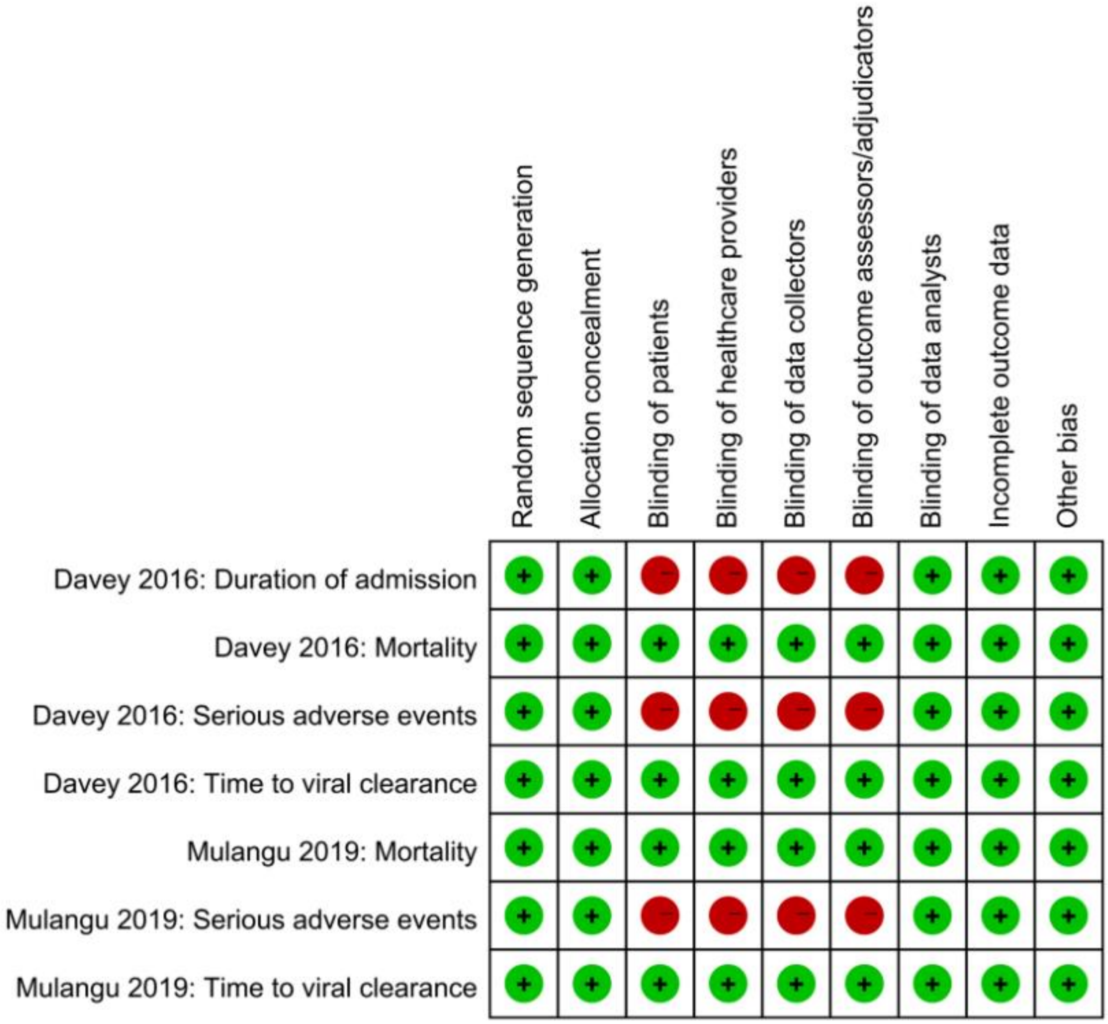

Note: Green represents low risk of bias; red represents high risk of bias.
